# A Notable Achievement

**Published:** 1909-05

**Authors:** 


### A Notable Achievement.

MR. Charles Gordon Hayd, president of the class of 1909, Medical Department University of Buffalo, to be graduated May 28, instant, recently took the examination for interne at the New York Postgraduate Hospital. Thirty candidates entered the arena, one having failed to appear before the contest began.

These young men represented nearly all the universities and other great medical schools of the land. At the end of the first day one-half the number had disappeared. The second day proved too much for one-half the remainder; while the third day left but four still "in the running." And behold, Mr. Hayd "led all the rest," having secured first place in an examination noted alike for its severity and fairness.

It should be remarked that Mr. Hayd had already passed, standing first on the list, the examination for interne at the Buffalo General Hospital. This place he resigned when the New York appointment was won. The JOURNAL extends its congratulations to Mr. Hayd, who is a nephew of Dr. Herman E. Hayd, the well known surgeon of Buffalo.

---

GREATER University of Buffalo grows apace. Through the efforts of vice-chancellor Norton, $27,000 have been added to the treasury and within the last few days the sum is further increased by more recent contributions. Let the good work go on.

---

THE health department is preparing to enforce the antispitting ordinance, which goes into effect on July 1st. Small yellow cards, bearing the words—"You are violating the law against spitting—you are subject to imprisonment or fine or both, by order of the health board—" are being printed ready for distribution.

The plan is to give these cards to school children, business-men and others, who, when they see a person spitting on the side-walk or in a public building or conveyance, are expected to hand one of the reminders to the offender. Other notices are being prepared for posting in public places.

---

ALLAN WILMOT, son of Mr. and Mrs. W. B. Wilmot, died March 31, 1909, of spinal meningitis. He was a student at the University of Buffalo and a graduate of the Delavan High School.

